# Estimating heterogeneous impacts Of subsidised health insurance: A causal machine learning approach

**DOI:** 10.1371/journal.pone.0315057

**Published:** 2025-09-29

**Authors:** Vishalie Shah, Noemi Kreif

**Affiliations:** 1 Centre for Health Economics, University of York, York, England, United Kingdom; 2 The CHOICE Institute, School of Pharmacy, University of Washington, Seattle, Washington, United States of America; Korea Institute for Pharmaceutical Policy Affairs, KOREA, REPUBLIC OF

## Abstract

The evaluation of social and health policies often necessitates understanding the variations in impacts based on recipients’ observed characteristics, underscoring the value of estimating treatment effect heterogeneity. In this study, we leverage predictive and causal machine learning to assess the impact of the subsidised component of Indonesia’s National Health Insurance Programme (“JKN”) on healthcare utilisation in 2017. We employ causal forests for estimating heterogeneous treatment effects and the super learner algorithm for prediction tasks. Our approach addresses the prevalence of zeros in the utilisation outcomes through a two-part model, which separates the outcome model into zero and non-zero counts. This allows for distinct investigation of policy impacts on the decision to seek care and the quantity of care consumed. We interpret and summarise treatment effect heterogeneity using various approaches, including data-driven subgroup analyses and linear projections, which are grounded in theory. Our results demonstrate a positive average impact on healthcare demand with evident heterogeneity; for instance, the increase in demand varies among recipients. We also find that the effect is modified by a set of theoretically motivated covariates and those identified through our data-driven approach.

## 1 Introduction

Universal health coverage is a policy priority in many low- and middle-income countries around the world, aimed at ensuring access to essential healthcare services without financial hardship [11,46]. Indonesia’s Jaminan Kesehatan Nasional (JKN), introduced in 2014, is the world’s largest single-payer health insurance system, with population coverage surpassing 80% by 2019 [51]. Prior to JKN, Indonesia’s health insurance system was fragmented, with separate schemes targeting specific subgroups: Askes for formal public sector workers, Jamsostek for formal private sector workers, and Jamkesmas for the poor. Informal workers, who make up 60% of the workforce, were largely excluded from these schemes [3,50]. JKN unified these schemes into a single national system, standardising benefits across socioeconomic groups to improve coverage equity [1,21].

JKN operates as a mandatory social health insurance program aiming for universal coverage of Indonesia’s entire population. The programme employs a dual financing mechanism: contributory for formal sector workers who pay income-based premiums, and non-contributory for the poor and near-poor whose premiums are fully subsidised by government revenue (known as JKN Penerima Bantuan Iuran (PBI) or Jamkesda). All citizens are eligible to enrol, with informal workers able to join through voluntary contributions at fixed premium rates. The program is administered by BPJS Kesehatan (Social Security Agency for Health), offering a comprehensive benefit package delivered through a tiered referral system from primary health facilities to specialised care when necessary. Despite these achievements, challenges remain, including slow enrolment among informal workers, persistent health disparities, and supply-side constraints, particularly in rural areas [1,50].

Evaluating the impact of large-scale health insurance programs like JKN is essential for understanding their effectiveness and equity implications. While most studies focus on estimating the overall average treatment effect (ATE) [27], exploring treatment effect heterogeneity – how impacts vary across population subgroups – is equally important. This is particularly relevant in Indonesia, where disparities in healthcare access and disease burden persist across geographic and socioeconomic lines [1]. Identifying which groups benefit most from JKN can provide valuable insights for improving its implementation and achieving UHC objectives.

Advances in machine learning have introduced powerful tools for estimating heterogeneous treatment effects (HTEs) in high-dimensional and complex datasets [6,7]. Traditional methods for exploring heterogeneity, such as regression-based interaction terms, often rely on restrictive parametric assumptions and face challenges like multiple hypothesis testing [4,23]. In contrast, causal machine learning approaches, such as causal forests, enable flexible, data-adaptive modelling of treatment effect heterogeneity across the entire covariate space while maintaining valid inference [8,17]. These methods combine predictive machine learning with the potential outcomes framework, offering researchers a robust approach to uncovering how treatment effects vary across different subgroups [57].

In this study, we employ predictive and causal machine learning methods to evaluate the impact of JKN on outpatient and inpatient healthcare utilisation. Given the prevalence of zero counts in the data, we adopt a two-part model to separately assess the effects on care-seeking decisions (participation) and the intensity of care consumed (consumption) [12,52]. Specifically, we estimate the average and heterogeneous effects of enrolment in the subsidised JKN scheme for the poor (JKN-PBI), compared to being uninsured. To achieve this, we leverage causal forests to estimate Conditional Average Treatment Effects (CATEs) and the super learner to estimate nuisance parameters, such as the outcome regression and propensity score [8,64]. These methods allow us to flexibly model treatment effect heterogeneity across a high-dimensional covariate space without relying on restrictive parametric assumptions [53,55].

Our analysis explores how treatment effects vary across demographic, geographic, and socioeconomic subgroups, using both theory-driven and data-driven approaches for selecting effect modifiers. We summarise treatment effect heterogeneity through approaches such as subgroup analysis, CATE-based population rankings, and linear projections to identify the most influential predictors of treatment effects [17,42,57]. This enables us to assess whether certain subgroups – such as rural residents, older individuals, or those with limited access to healthcare infrastructure – derive greater or lesser benefits from subsidised JKN enrolment.

We conduct this evaluation using data from the 2017 National Socioeconomic Survey (SUSENAS), a nationally representative, repeated cross-sectional dataset collected at the household-member level [38]. SUSENAS provides detailed information on health care utilisation, demographics, and socioeconomic factors, allowing us to control for observed confounding and explore treatment effect heterogeneity in a robust manner. By integrating predictive and causal machine learning methods, this study contributes to the growing literature on the evaluation of UHC policies, offering actionable insights for improving the equity and effectiveness of health insurance programs in Indonesia and beyond.

## 2 Methods

### 2.1 Data

We use data from the 2017 National Socioeconomic Survey (SUSENAS), conducted by Indonesia’s central statistics agency, Badan Pusat Statistik [38]. This nationally representative survey collects data annually from approximately 300,000 households, comprising 1.1 million household members across all 34 provinces and 514 districts. SUSENAS employs a two-stage sampling design to ensure representative household selection at the district level, with frequency weights reflecting the national population [38].

For this analysis, we extract two key measures of healthcare utilisation: (1) the number of outpatient visits in the past month (a count variable) and (2) the total length of inpatient stays (in days) in the past year. We selected the 2017 wave of SUSENAS because it was the most recent dataset available at the time of analysis that included the outpatient utilisation variable, which is essential to our two-part model structure but was not present in later waves. The longer recall period for inpatient care reflects its relative rarity compared to outpatient care [12]. We consider treatments received at both public and private medical facilities, excluding traditional or alternative medical treatments. These measures are divided into two components: a binary indicator of treatment presence (termed “participation") and a count variable capturing the intensity of treatment among those who sought care (termed “consumption"). This structure forms the basis for our subsequent two-part modelling approach.

Our focus is on respondents reporting either “no insurance" or enrolment in subsidised health insurance schemes, specifically JKN-PBI or Jamkesda. We exclude respondents with additional health insurance plans, following formal guidelines, as they constitute only 2.5% of the overall sample. The final dataset consists of 912,812 household members across 297,276 households, with 475,930 members in the subsidized “treated" group and 436,882 in the uninsured “control" group.

We construct a comprehensive covariate vector (*X*), incorporating 89 variables to account for confounding factors and potential effect modifiers. These variables are selected based on theoretical reasoning and prior studies on similar health insurance programs in Indonesia [27,66]. The covariates (see Appendix A for a comprehensive list) include:

- Demographic factors: Age, sex, marital status.
- Socioeconomic indicators: Education, employment status, household expenditure, and technology usage (e.g., internet access and usage within the past three months).
- Housing characteristics: Asset ownership, access to basic facilities like electricity and clean water.
- Geographic factors: Urban/rural residence, regional dummies.
- Healthcare access indicators: Derived from the 2018 PODES village-level census data, which provide information on access to hospitals, primary care facilities, community health centers, and maternity services. Accessibility is categorised into binary indicators based on ease of access (easy/very easy vs. difficult/very difficult).

Our final dataset consists of 912,812 household members across 297,276 households, with 475,930 members in the subsidised ‘treated’ group and 436,882 in the uninsured ‘control’ group. To ensure the findings are generalisable to Indonesia’s population, sample weights are applied throughout the analysis.

### 2.2 The causal framework

We construct an observational dataset $\left(X_{i},Y_{i},D_{i}\right)$ of household members i,…,N, where *X*_*i*_ is the vector of confounders and potential effect modifiers, *D*_*i*_ is the binary treatment (which equals 1 if *i* is enrolled into subsidised JKN, and 0 if *i* is uninsured), and *Y*_*i*_ are the continuous outcomes that measure the utilisation of inpatient and outpatient health care. Following the potential outcomes framework of causal inference [35,56], *Y*_*i*_(*d*) denotes the potential outcome that would be observed if household member *i* was assigned to treatment *d*. Individual level treatment effects are defined as the difference in the potential outcomes: $\tau_{i}=Y_{i}(1)\;-\;Y_{i}(0)$. However, given the fundamental problem of causal inference, $\tau_{i}$ cannot be observed. We can instead take expectations of the difference in the potential outcomes across the population to produce the ATE: $\tau =E[Y_{i}(1)\;-\;Y_{i}(0)]$.

Since we are interested in exploring the variation in treatment effects across the population, we also define the CATE function, which evaluates the ATE for individuals with the same covariate profile *X*_*i*_ = *x*:

$$\tau (x)=E[Y_{i}(1)\;-\;Y_{i}(0)|X_{i}=x],$$ (1)

This function captures heterogeneity in treatment effects through effect modifiers included in *X*. The ATE can also be defined as the expectation of the CATE function over a population represented by the distribution of *X*, $\tau =E_{X}[\tau (x)]$.

The CATE is our target causal parameter, which in order to be identified using the observed data, requires making the following assumptions on the data generating process. First, the unconfoundedness assumption (also known as selection on observables): $\{Y_{i}(0),Y_{i}(1)\}⟂D_{i}|X_{i}$, which requires the potential outcomes to be independent of treatment status, conditional on the observed covariates. Second, the overlap assumption: $0<e(X_{i})\equiv P(D_{i}=1|X_{i}=x)<1$, which requires the probability of being enrolled into subsidised JKN (that is, the propensity score *e*(*x*), which we also refer to as the treatment model) to be bounded away from zero and one. If these two assumptions (jointly referred to as strong ignorability) are satisfied, the conditional expectation of the potential outcomes equals the conditional expectation of the observed outcome. The CATE can therefore be identified as a function of the observed outcomes:


$$\tau (x)=E[Y(1)|X_{i}=x]-E[Y(0)|X_{i}=x]$$

$$=E[Y_{i}|X_{i}=x,D_{i}=1]-E[Y_{i}|X_{i}=x,D_{i}=0],$$

where $E[Y(1)|X_{i}=x]$ and $E[Y(0)|X_{i}=x]$ are the counterfactual response surfaces, and $E[Y_{i}|X_{i}=x,D_{i}=1]$ and $E[Y_{i}|X_{i}=x,D_{i}=1]$ are their counterparts that can be estimated from the data, given the causal assumptions.

We also define $m(x)=E[Y_{i}|X_{i}=x]$, the conditional expectation of the outcome given covariates, but pooled across treatment groups.

Estimating CATEs with causal forests relies on good estimates of *m*(*x*) and *e*(*x*), which are collectively referred to as the so-called nuisance models that are required for estimating the target causal parameter but are not directly of interest. These models are essentially prediction tasks, allowing for flexible model specifications using machine learning instead of traditional parametric approaches [36].

### 2.3 Descriptive analysis

We conducted a descriptive analysis to summarise the baseline characteristics of the insured (treated) and uninsured (control) groups and to assess covariate balance between the groups. Means and standard deviations were calculated for continuous variables (e.g., age, household expenditure), while proportions were reported for categorical variables (e.g., sex, marital status, healthcare access indicators). To assess baseline differences, we computed standardised mean differences (SMDs) for all covariates, with an absolute SMD < 0.1 indicating acceptable balance.

As an initial assessment of the ability of our subsequent statistical analysis to adjust for observed confounding, we applied inverse probability of treatment weighting (IPTW) based on estimated propensity scores. IPTW reweights the sample to balance the distribution of covariates across treatment groups, simulating a pseudo-population where the covariates are independent of treatment assignment. Specifically, we use ATE weights that re-weight both the insured and uninsured groups to resemble the composition of the entire study population (see Sect 2.4 for details on the propensity score estimation). The results of the descriptive analysis, including pre- and post-weighting characteristics, are reported in Table 2, providing a basis for the causal analysis. We note that while the subsequent analysis utilises the propensity score for confounding adjustment, it does so in a slightly different form, and also exploits outcome regression models for double robust confounding adjustment (see details in Sects 2.4, 2.5, and 2.6).

### 2.4 Estimating nuisance parameters using the super learner

We use the super learner to estimate *e*(*x*) and *m*(*x*), leveraging various machine learning techniques such as ensembling and *K*-fold cross-validation [14,63,64]. The super learner ensemble combines heterogeneous “base" learners into an optimally-weighted ensemble, enhancing model accuracy. It uses *K*-fold cross-validation to evaluate the performance of base learners by iteratively partitioning the sample into *K* folds, then making predictions on each validation fold *k* after training the base learners on the remaining *K*–*k* folds. Model performance is evaluated by calculating the average loss, such as mean squared error (MSE), across all validation folds using predicted and observed outcomes. Additionally, the super learner incorporates a metalearner to determine optimal weights for combining the predictions of the base learners.

For the treatment model, we select base learners designed for binary outcomes, while for the outcome model, we adopt a two-part framework to handle the significant mass points at zero in our outcome distributions. This framework acknowledges that care access stratifies the population into users and non-users, with care consumption largely influenced by supply-side factors [54]. Two-part models, commonly employing logit or probit models for the binary “participation" component and poisson or negative binomial models for the truncated-at-zero count “consumption" component [52], are popular for modeling healthcare utilisation due to their ability to account for these separate processes. We propose using two super learners to predict the distinct components of the two-part model:

$$m(x)=E[Y_{i}|X_{i}]=\underset{m_{1}(x)}{\underbrace{P(Y_{i}>0|X_{i}=x)}}\cdot \underset{m_{2}(x)}{\underbrace{E[Y_{i}|Y_{i}>0,X_{i}=x]}},$$ (2)

where *m*_1_(*x*) and *m*_2_(*x*) are the respective participation and consumption components.

Here, we outline the super learner procedure for predicting the consumption component of the outcome model *m*_2_(*x*) (a similar procedure applies for all other prediction tasks, with the loss function adjusted based on outcome type (e.g. log likelihood loss for binary outcomes, MSE for continuous outcomes). We select a diverse set of base learners, including both parametric and non-parametric models (see Table 1 for a full list). Linear models are included but can receive a low weight if they do not fit the data well, as determined by the ensemble learner. To adjust for confounding, we include the full covariate vector *X*. Performance is compared against conventional hurdle models, which use logistic regression for the participation component and truncated-at-zero Poisson and negative binomial models for the consumption component. Cross-validation with *K* = 5 folds is performed to assess model performance and generate predictions ${\hat{Y}}_{l}$ for each learner l=1,…,L. These predictions, alongside the observed outcomes *Y*, are then input into a metalearner that optimises a linear combination of base learners through stacking. We use ordinary least squares (OLS) for the meta-learner instead of non-negative least squares (NNLS), allowing weights that do not sum to one due to the inclusion of a constant term and absence of non-negativity constraints. Finally, another *K*-fold cross-validation evaluates the ensemble learner’s performance against individual base learners, resulting in cross-validated nuisance predictions.

**Table 1:** Candidate algorithms included in the super learner libraries.

Algorithm	Description	Binary	Count
glm-g	Generalised linear model (gaussian)		x
glm-b	Generalised linear model (binomial)	x	
glm-p	Generalised linear model (poisson)		x
glm-nb	Generalised linear model (negative binomial)		x
lasso-g	LASSO (gaussian)		x
lasso-b	LASSO (binomial)	x	
lasso-p	LASSO (poisson)		x
lasso-nb	LASSO (negative binomial)		x
rf	Random forest	x	x
gbm	Generalised boosting model	x	x
nn	Neural network	x	x

*Note:* Table shows the algorithms included in the respective super learner libraries for binary and count tasks. Binary tasks include the estimation of the treatment model and the participation components of the outcome models. Count tasks include the estimation of the consumption components of the outcome models.

**Fig C.1:**
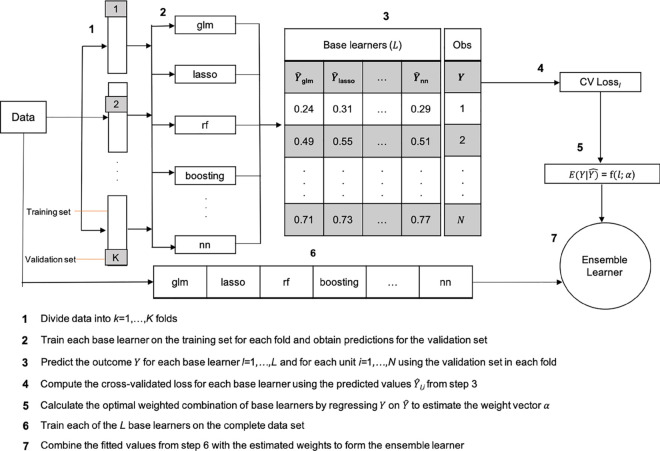
Super learner workflow.

See Fig C.1 for a super learner workflow.

### 2.5 Estimating conditional average treatment effects using causal forests

Causal forests extend the random forests algorithm, a popular technique for predictive modeling, to handle causal inference tasks [8]. In a causal forest, an ensemble of causal trees is constructed through recursive partitioning of the data, aiming to identify distinct groups of observations with similar treatment effects. Unlike traditional random forests, where the splitting criterion minimises prediction error, causal forests search for partitions that maximise heterogeneity in treatment effects across leaves. This means that within each partition, treatment effects are similar, while they vary across partitions. Importantly, causal forests employ “honesty" to prevent overfitting, meaning that each observation is either used to construct the tree structure or to estimate the treatment effect within a leaf, but not both. This helps to ensure that the model generalises well to unseen data. The ensemble approach of causal forests addresses the inherent instability of individual trees by aggregating predictions from multiple trees, thereby reducing variance.

Following tree construction, the CATE function τ(x) is estimated using weights derived from all trees in the forest, as follows:

$$\hat{\tau }(x)=\frac{\sum_{i=1}^{N}w_{i}(x)(D_{i}-\hat{e}(X_{i}))(Y_{i}-\hat{m}(X_{i}))}{\sum_{i=1}^{N}w_{i}(x)(D_{i}-\hat{e}(X_{i}){)}^{2}},$$ (3)

where $\hat{m}(X_{i})$ and $\hat{e}(X_{i})$ are the estimated nuisance parameters, and $0\leq w_{i}(x)\leq 1$ are weights for each observation based on their similarity to a specific covariate profile *x*. The weights are determined based on the frequency of observations sharing the same leaf as a given observation with covariate profile *x* across all trees in the forest.

See Appendix B for further details on the causal forest algorithm.

### 2.6 Summarising and interpreting treatment effect heterogeneity

When analysing predictions of CATEs, it’s crucial to recognise that they alone may not provide clear interpretability. Instead, our focus should be on discerning whether significant heterogeneity exists and identifying which covariates drive this heterogeneity.

One common strategy advocated in the literature is to construct almost unbiased, albeit noisy, proxies for the predicted CATEs, known as doubly robust scores $\Gamma_{i}$. We derive these scores using the doubly robust estimator for the Augmented Inverse Probability of Treatment Weighted (AIPTW) estimator for the ATE, defined as:

$$\hat{\tau }=\frac{1}{N}\sum_{i=1}^{N}{\hat{\Gamma }}_{i}(X_{i}),\;{\hat{\Gamma }}_{i}=\hat{\tau }(X_{i})+\frac{D_{i}-\hat{e}(X_{i})}{\hat{e}(X_{i})(1-\hat{e}(X_{i}))}(Y_{i}-\hat{m}(X_{i})-(D_{i}-\hat{e}(X_{i})\hat{\tau }(X_{i})).$$ (4)

The doubly robust scores can be used to examine treatment effect heterogeneity, as detailed in the following sections.

#### Assessing heterogeneity

*Reporting Group ATEs based on CATE Quintiles*: Individuals are categorised into quintiles based on their estimated CATEs, with Q1 representing the lowest predicted treatment effects and Q5 the highest. Average Treatment Effects (ATEs) are then estimated for each quintile using doubly robust scores derived from the AIPTW estimator in (5) [9]. To ensure that observed heterogeneity reflects true treatment effects rather than confounding, the construction of CATE quintiles relies on these doubly robust scores. These quintile-based ATEs, also known as “sorted group ATEs” (GATEs), help identify significant differences in treatment effects across population subgroups [17].

#### Identifying drivers of heterogeneity

*Variable Importance Analysis*: Within the context of causal forests, variable importance analysis quantifies the contribution of each covariate to predicting treatment effects. This method ranks covariates based on their overall predictive importance but does not provide insights into the direction or magnitude of their effects on the CATE. Additionally, continuous covariates are often favoured due to their wider range of variability, which may influence their ranking.

*Classification Analysis based on CATE Quintiles*: Classification analysis enables systematic comparisons of baseline covariates across subgroups with varying treatment effects. Individuals are categorised into quintiles based on estimated CATEs, and means and proportions of demographic, socioeconomic, geographic, and healthcare access covariates are summarised for each quintile. Differences between quintiles are quantified using SMDs, with larger SMDs indicating greater heterogeneity. Heatmaps are used to visualise relative differences in covariate distributions across quintiles, providing an intuitive representation of how specific characteristics vary across individuals with differing levels of predicted treatment effects.

*Subgroup Analysis*: Subgroup analysis identifies distinct population subsets that show varying treatment effects. Subgroups are defined based on key covariates, such as age, sex, household expenditure, and healthcare access, selected through prior literature or exploratory analysis. Group Average Treatment Effects (GATEs) are estimated for each subgroup using AIPTW applied to restricted samples [44]. This approach provides insights into how treatment effects differ across segments such as rural versus urban residents or individuals with varying healthcare accessibility. While subgroup analysis is useful for understanding heterogeneity, it does not account for interactions between covariates and may lack a ceteris paribus interpretation. Nonetheless, it offers valuable context for identifying population groups that benefit most or least from the intervention.

*Best Linear Projection (BLP) Analysis*: As described by [57], BLP analysis projects predicted CATEs onto a simpler hypothesis space to provide a ceteris paribus interpretation of treatment effect heterogeneity. This method estimates the relationship between covariates and treatment effects using OLS regression of doubly robust scores on selected covariates, with standard errors computed conventionally. Covariates are selected based on theory-driven and data-driven approaches, focusing on demographic factors (e.g., age, sex), socioeconomic indicators (e.g., household expenditure, education), and healthcare access variables. While BLP enables inference on the “effect" of each covariate on the expected CATE, caution is warranted in interpreting coefficients as partial effects, as relationships may be nonlinear. Despite this limitation, BLP provides a rigorous framework for identifying key predictors of heterogeneity.

#### Ensuring robustness

All methods for examining treatment effect heterogeneity rely on sample-splitting and cross-fitting to mitigate bias and overfitting. Sample-splitting divides the dataset into separate subsets for estimating nuisance parameters (e.g., propensity scores and outcome regressions) and treatment effects, ensuring independence between model training and testing [42]. Cross-fitting, such as the K-fold cross-fitting approach proposed by [18], further enhances robustness by swapping roles of training and testing subsets, generating out-of-sample double robust scores. These techniques reduce the risk of overfitting while maximising statistical efficiency. By leveraging cross-fitted scores, subsequent analyses, such as subgroup analysis or the identification of linear predictors of the CATE function, can be conducted on the full dataset, ensuring that results are reliable and generalisable.

### 2.7 Our implementation

Our implementation of the methods outlined is tailored to address our health insurance policy evaluation problem. We conduct analyses on two outcome measures: inpatient and outpatient demand, employing three distinct outcome models: the overall two-part model, the participation component, and the consumption component.

**Fig 1:**
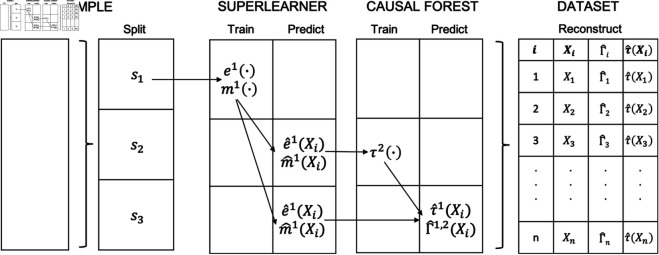
Cross-fitting workflow. *Note:* This visual aid presents a single round of the cross-fitting process using three subsamples: *s*_1_, *s*_2_, and *s*_3_. In this round, *s*_1_ is used as the training set for the propensity score model $e^{1}(\cdot )$ and the outcome model $m^{1}(\cdot )$. These trained models are then applied to *s*_2_ to generate the propensity score and outcome model estimates, which are subsequently used as inputs for the CATE model ${\hat{\tau }}^{2}(\cdot )$. Final CATE predictions and doubly robust scores are then computed for *s*_3_, the test sample in this round. The subscripts on the estimated parameters denote the subsample used for training. Note that this figure illustrates only one round. In the full cross-fitting process, the training sample will rotate amongst *s*_2_ and *s*_3_ in subsequent rounds, allowing the use of estimated parameters from each round to reconstruct the complete dataset.

Our implementation proceeds as follows:

1. We construct the dataset and identify potential theory-driven effect modifiers from the covariate vector *X*, guided by subgroup analyses in the literature. For the CATE models, all variables are considered effect modifiers, including demographic, socioeconomic, geographic and supply-side factors such as age, household wealth (measured by per capita monthly consumption), urban vs. rural location, and local health care facility availability [27].
2. Using a 3-fold cross-fitting approach, the data is divided into three equal subsets: *s*_1_, *s*_2_, and *s*_3_:
  - Subset *s*_1_ is designated for training the nuisance models (m(·) and e(·)) using super learners implemented with the h2o package [34]. These models are fitted to *s*_1_, and predictions are made for *s*_2_ and *s*_3_.
  - Subset *s*_2_ is used for training the CATE function (τ(·) via a causal forest from the grf package [31]. An honest causal forest is trained on *s*_2_ using observed and predicted inputs from *s*_1_. Default parameters, including 2000 trees for training and 200 trees for tuning, are selected alongside sample weights (see Table C.1 for further details).
  - Subset *s*_3_ is used to construct doubly robust scores ${\hat{\Gamma }}_{i}$ (as in 5), which requires generating predictions of the nuisance functions ($\hat{m}(X_{i})$, $\hat{e}(X_{i})$) and the CATE function ($\hat{\tau }(X_{i})$) using the trained models from *s*_1_ and *s*_2_, respectively.
3. The cross-fitting process is repeated twice, alternating the roles of the subsets to ensure robustness. This generates out-of-sample predictions, $\hat{\tau }(X_{i})$ and ${\hat{\Gamma }}_{i}$, for each subset, facilitating the reconstruction of the full dataset. See Fig 1 for a visual demonstration of our cross-fitting workflow.
4. The pooled data is divided into quintiles (Q1-Q5) based on the estimated CATEs $\hat{\tau }(X_{i})$. Subsequent subgroup analyses are conducted as follows:
  - Using the predicted doubly robust scores ${\hat{\Gamma }}_{i}$ constructed from the predicted nuisance and CATE functions ($\hat{m}(X_{i})$, $\hat{e}(X_{i})$) and ${\hat{\tau }}_{i}$), we estimate sorted GATEs for each quintile and overall sample ATEs, using the AIPTW estimator.
  - To assess the significance of treatment effect differences between quintiles, we employ a difference-in-means estimator, incorporating the Romano-Wolf correction for multiple hypothesis testing [19].
  - We perform classification analysis that summarises the joint distribution of covariates across quintiles by comparing quintile-specific covariate means to overall covariate means, using heatmaps. We identify the top five covariates with the largest difference in standardised means between Q1 and Q5 as “data-driven" effect modifiers, contrasting with effect modifiers derived from the variable importance ranking of the causal forest.
5. For subgroup analyses, we use the AIPTW estimator to construct GATEs based on predefined and data-driven effect modifiers. Continuous variables are dichotomised for interpretation.
6. We conduct BLP analysis by regressing the doubly robust scores ${\hat{\Gamma }}_{i}$ on covariates *X*_*i*_, incorporating both the full covariate vector and a restricted vector of theory- and data-driven effect modifiers. To facilitate interpretation, continuous variables are dichotomised. The estimated coefficients (with their standard errors) in the linear model are used to make inferences about drivers of treatment effect heterogeneity.

## 3 Results

### 3.1 Descriptive statistics

Table 2 presents the descriptive statistics for the insured (treated) and uninsured (control) groups. The reported unweighted means and standard deviations reflect the raw differences between the two groups. To address potential differences in observed covariates, weighted standardised mean differences using IPTW weights are also presented in this table, as described in Sect 2.3. In this section, we compare the covariate balance for the raw (unweighted) sample. The weighted balance statistics are presented in the following section, and also in Fig C.3.

While most covariates in *X* show similarities between the two groups, some small differences exist (with absolute standardised mean differences (SMD) > 0.1). The insured population tends to be older with a mean age of 31 compared to 27 for the uninsured. In terms of geographic distribution, the insured are more prevalent in Sumatera (25% vs. 21%), Jakarta (4% vs. 2%), Sulawesi (10% vs. 7%), and Maluku-Papua (4% vs. 3%) compared to the uninsured. Regarding socioeconomic characteristics, 63% of the insured have completed compulsory education, compared to 55% of the uninsured, and literacy rates are higher among the insured (87% vs. 79%). The insured also reside in larger households, with an average household size of 5.3 members compared to 5.2 for the uninsured, but report lower monthly per capita expenditures (IDR 781,500 vs. IDR 847,000). Access to healthcare facilities also differs between the groups. For instance, 79.6% of the insured report “easy" or “very easy" access to primary healthcare facilities, compared to 81.9% of the uninsured. As expected, healthcare utilisation is higher among the treated group, with 12.8% of the insured reporting outpatient visits in the past month compared to 11.2% of the uninsured, and 4.3% of the insured reporting inpatient stays in the past year compared to 2.6% of the uninsured.

### 3.2 Average impacts of health insurance

#### 3.2.1 Performance of prediction algorithms.

Table C.2 summarises the performance of the super learners, including the weighting of individual base learners and their cross-validated loss. The super learner achieves optimal performance across most components, demonstrating its ability to handle complex data structures. For the propensity score model, the ensemble learner achieves a log loss of 0.550, with the generalised boosting model receiving the largest weight in the ensemble. For the inpatient participation model, the ensemble achieves a loss of 0.142, with the generalised boosting and random forest models contributing most heavily. In the inpatient consumption model, the ensemble achieves a loss of 2.545, with the generalised linear model (Gaussian) contributing the most weight. For outpatient models, the ensemble achieves a participation loss of 0.342 and a consumption loss of 0.376, again outperforming individual base learners. Notably, the super learner also outperforms the separate components of the conventional two-part hurdle model across all tasks, demonstrating its robustness and flexibility in predicting both participation and consumption outcomes.

**Table 2:** Descriptive statistics.

	Uninsured		Insured		SMD	
	Mean	SD	Mean	SD	Raw	IPTW
Outcomes						
Had inpatient treatment	0.026	0.158	0.043	0.203	0.018	0.023
Total length of inpatient stay in past one year (days)	0.121	1.151	0.247	1.972	0.078	0.095
Had outpatient treatment	0.112	0.316	0.128	0.334	0.015	0.026
Total number of outpatient visits in past one month	0.160	0.563	0.195	0.687	0.056	0.073
[0.3em] Household member characteristics						
Male	0.508	0.500	0.503	0.500	-0.005	-0.003
Age	26.7	19.8	30.5	19.5	0.197	-0.012
Education: compulsory	0.554	0.497	0.629	0.483	0.075	-0.006
Education: non-compulsory	0.236	0.424	0.248	0.432	0.013	0.000
Literate: Latin letters	0.789	0.408	0.871	0.335	0.082	-0.004
Employment status: in employment	0.403	0.490	0.455	0.498	0.053	0.001
Employment status: student	0.153	0.360	0.180	0.384	0.026	-0.002
Marital status: married	0.433	0.495	0.482	0.500	0.050	0.001
Used internet in previous 3 months	0.241	0.428	0.239	0.426	-0.003	0.004
Travelled domestically for tourism in 2016	0.237	0.425	0.218	0.413	-0.019	0.007
[0.3em] Household characteristics						
Location: urban	0.483	0.500	0.459	0.498	-0.024	-0.008
Number of people in household	5.191	2.017	5.282	2.007	0.045	-0.020
Home occupancy status: owner	0.834	0.372	0.850	0.358	0.016	0.006
Has a second home	0.092	0.289	0.115	0.318	0.022	-0.009
Toilet: private	0.876	0.329	0.829	0.376	-0.047	0.010
Purchases drinking water	0.431	0.495	0.411	0.492	-0.020	-0.002
Electricity	0.982	0.133	0.970	0.171	-0.012	0.000
Goods ownership: car	0.102	0.303	0.067	0.250	-0.036	0.004
Natural disaster in previous year	0.112	0.316	0.138	0.345	0.026	-0.006
Not enough food to eat in previous year	0.249	0.433	0.291	0.454	0.042	-0.013
Has a savings account	0.461	0.498	0.432	0.495	-0.029	0.008
Monthly consumption expenditure per capita (IDR 100,000)	8.469	7.194	7.815	6.384	-0.096	0.025
[0.3em] Health care accessibility						
Easy access: secondary care	0.757	0.205	0.741	0.221	-0.076	0.015
Easy access: community care	0.920	0.114	0.913	0.127	-0.059	-0.001
Easy access: primary care	0.819	0.179	0.796	0.213	-0.116	0.030
Easy access: maternal care	0.854	0.176	0.840	0.203	-0.075	-0.001
[0.3em] Region						
Region: Sumatera	0.213	0.409	0.250	0.433	0.038	0.005
Region: Jakarta	0.019	0.137	0.039	0.194	0.020	-0.007
Region: Java	0.536	0.499	0.458	0.498	-0.078	0.017
Region: Bali,NTB,NTT	0.064	0.244	0.062	0.242	-0.001	-0.006
Region: Kalimantan	0.073	0.260	0.046	0.210	-0.026	0.003
Region: Sulawesi	0.070	0.255	0.099	0.299	0.030	-0.011
Region: Maluku-Papua	0.026	0.159	0.044	0.206	0.018	0.000

*Note:* Sample means and standard deviations (SD) are reported for selected variables in *X* for the uninsured and insured (enrolled into subsidised JKN) populations. SMD = standardised mean difference. Raw = unweighted. IPTW = inverse probability of treatment weighting for the ATE. All observations are weighted by SUSENAS frequency weights.

**Fig C.2:**
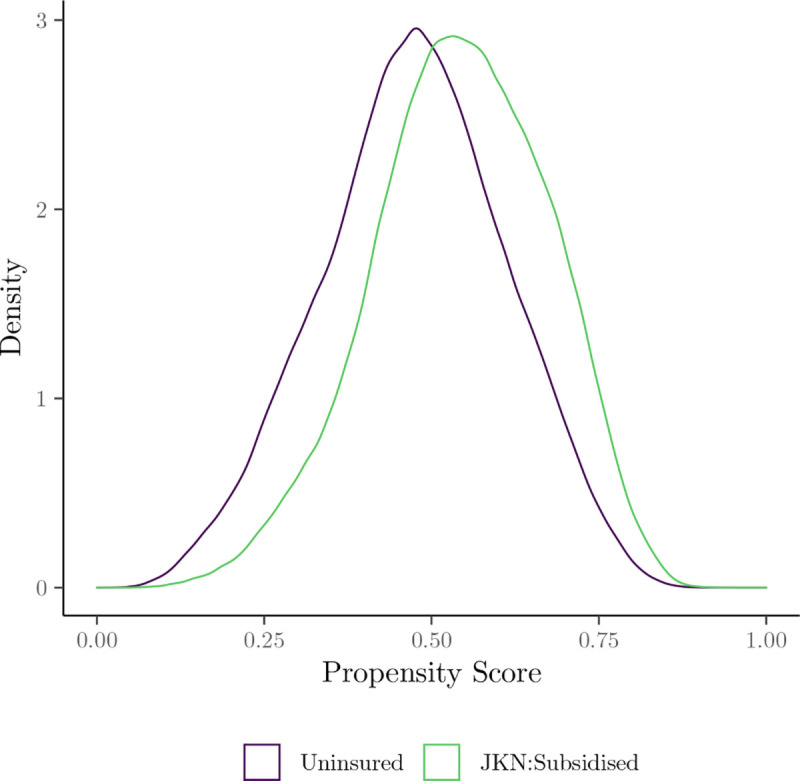
Overlap plot. *Note:* Density plot showing the distribution of predicted propensity scores for the treated (subsidised JKN) and the controls (uninsured) in the pooled data.

**Fig C.3:**
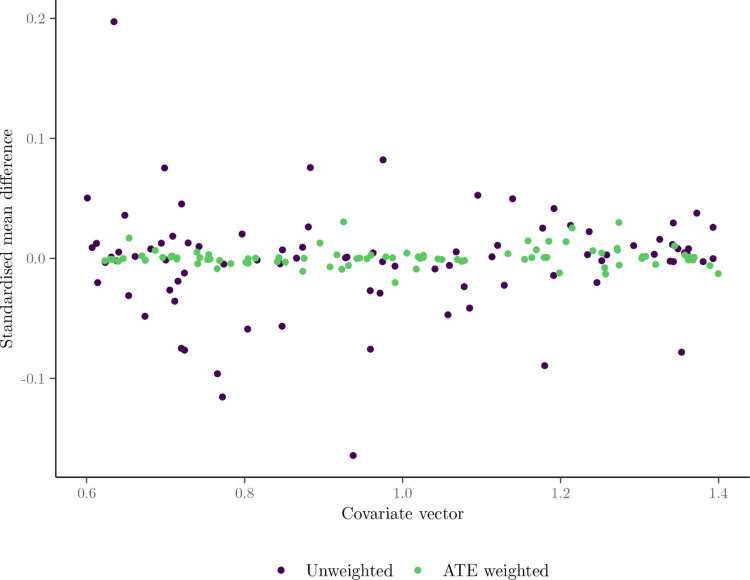
Covariate balance between the subsidised JKN and the uninsured populations. *Note:* Standardised mean differences are reported for all X between the treated (subsidised JKN) and the controls (uninsured) before and after inverse probability of treatment weighting for the ATE.

Fig C.2 illustrates the distribution of predicted propensity scores, confirming satisfaction of the overlap assumption with no extreme scores close to 0 or 1. Fig C.3 presents balance statistics on the full covariate vector *X* before and after inverse probability of treatment weighting. Post-weighting, all covariates achieve an absolute SMD below 0.1, indicating successful adjustment for observed confounding.

#### 3.2.2 Average treatment effects.

**Fig 2:**
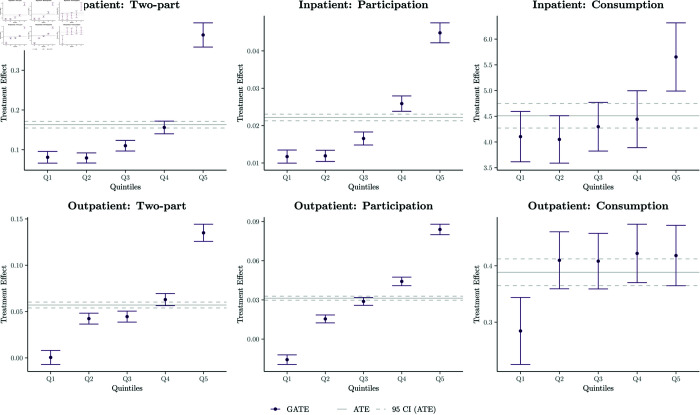
Estimated sorted GATEs. *Note:* Point estimates and 95% confidence intervals (error bars) are reported. GATEs are the estimated ATEs for each quintile of the population, ranked (in ascending order) by predicted CATEs. ATE point estimates and 95% confidence intervals are also reported. Graphs are separated by type of health care utilisation (inpatient and outpatient) and outcome model (two-part, participation component and consumption component).

Fig 2 presents the estimated ATEs and their corresponding 95% confidence intervals for the two-part, consumption, and participation models. Specifically, subsidised insurance leads to an average increase of 0.16 days in inpatient stays and 0.06 additional outpatient visits. While the effects on participation are modest, with an increase of 0.02 visits for inpatient care participation and 0.03 visits for outpatient care participation, the impact on consumption is more pronounced. Insured individuals who access care report an average increase of 4 days in inpatient stays and 0.4 additional outpatient visits compared to the uninsured.

These results indicate that subsidised insurance has a greater effect on the intensity of care consumed (consumption) than on the likelihood of accessing care (participation). This suggests that the program effectively reduces financial barriers for those already seeking care, enabling them to consume more healthcare services.

### 3.3 Heterogenous impacts of health insurance

#### 3.3.1 Testing for heterogeneity.

Fig 2 and Table C.3 provide evidence of treatment effect heterogeneity across the three models of interest: the two-part, participation, and consumption models. Substantial variation is observed in the effects of insurance on both inpatient and outpatient demand when stratified by ranked CATE-based quintiles. For inpatient demand, the two-part model estimates an additional increase of 0.31 days in Q5 compared to Q1, while the participation model shows an additional 3.3% increase in the likelihood of an inpatient stay for Q5 versus Q1. The consumption model highlights even greater heterogeneity, with insured individuals in Q5 reporting 1.55 additional increase in inpatient days compared to Q1 (adjusted *p*-value < 0.001). Significant differences are also observed between Q1 and Q4 across the two-part and participation models, indicating that heterogeneity is not limited to the most extreme quintiles.

For outpatient demand, the two-part model estimates an additional increase of 0.14 visits in Q5 compared to Q1, and the participation model shows an additional 10% increase in the likelihood of an outpatient visit for Q5 versus Q1. Significant differences are detected between Q1 and all other quintiles (Q2–Q5) across all models, suggesting a strong and consistent pattern of heterogeneity. Of particular interest is the negative impact on outpatient demand observed in Q1, where individuals experience lower or no gains in utilisation. This may be attributable to supply-side constraints, such as limited access to healthcare facilities, or a preference for inpatient care within this subgroup.

These results demonstrate that the impact of subsidised insurance varies significantly across population subgroups, with the largest benefits accruing to individuals in higher CATE quintiles (Q4–Q5). In contrast, individuals in Q1 show much smaller gains, particularly for outpatient care, highlighting the importance of addressing structural barriers to healthcare access for the least advantaged groups.

#### 3.3.2 Drivers of heterogeneity: Classification analysis.

**Fig 3:**
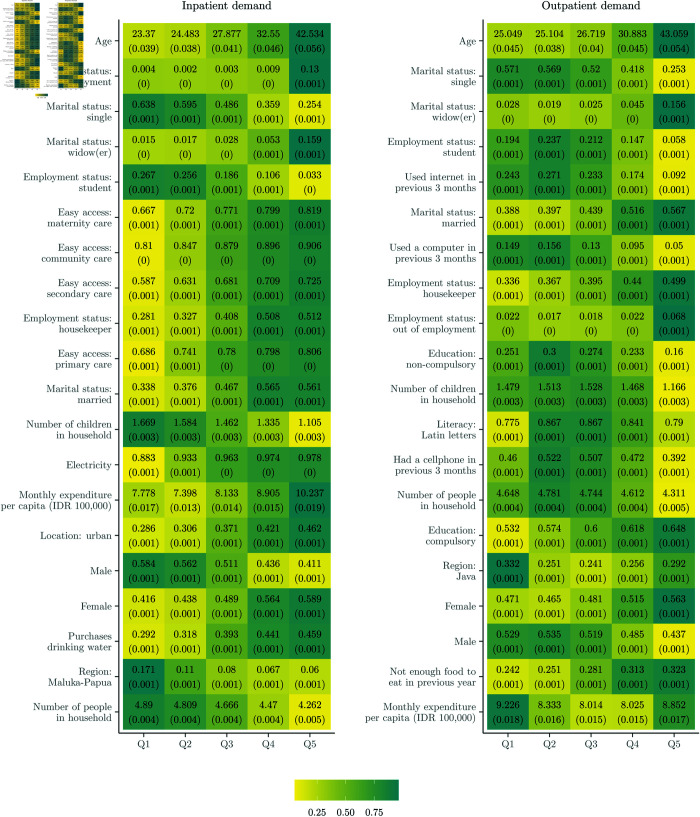
Heatmaps for classification analysis (two-part model). *Note:* Heatmaps show the variation in the joint distribution of covariates for subgroups with different CATEs. The annotated text shows the average value and standard error (in parentheses) of each covariate within each quintile (of predicted CATEs). The colour is a normalised distance of each quintile-specific covariate mean from the overall covariate mean. The darker the colour, the greater the distance. Only the top 20 covariates in *X* are plotted, in descending order of variation between Q1 and Q5.

Figs 3 and C.4 provide deeper insights through heatmaps illustrating the distribution of covariates across predicted CATE quintiles. Notably, considerable variation in covariate means is observed across CATE quintiles for all inpatient models, indicating diverse impacts of health insurance on inpatient demand based on individual characteristics. Key variables showing substantial variation include age, sex, accessibility to various healthcare facilities (such as maternity, community, secondary, and primary), and household expenditure.

Healthcare accessibility emerges as a critical driver of heterogeneity. In Q5, 80% of individuals report “easy" or “very easy" access to primary care facilities, compared to 69% in Q1. Similarly, access to secondary care facilities is reported as easy by 72% of Q5 individuals, compared to 59% in Q1. Policy effects are notably stronger among older individuals (mean age of 42.5 years in Q5 versus 23.4 years in Q1), non-single (56% married in Q5 versus 33% in Q1), and non-working individuals (13% “out of employment" in Q5 versus 0.4% in Q1) with better household amenities (98% with electricity in Q5 versus 88% in Q1) and higher expenditure (approximately IDR 1,000,000 in Q5 versus IDR 800,000 in Q1), particularly in urban areas (46% in Q5 versus 29% in Q1). Geographical heterogeneity is also evident, with 17% of residents in Maluku-Papua falling within the least affected quintile.

Regarding outpatient demand, age emerges as a prominent variable, with older individuals deriving greater benefits from health insurance, particularly in terms of increased care consumption. The average age in Q5 is 43 years, compared to 25 years in Q1. Women are overrepresented in Q5 for outpatient care, comprising 56% compared to 47% in Q1. A similar trend is observed for inpatient care, where women account for 59% of Q5, compared to 41% in Q1.

Additionally, previously unspecified variables such as marital status, food sufficiency, and technology ownership emerge as potentially important effect modifiers. In Q5, only 40% report recent cellphone usage compared to 46% in Q1, and 9% have used the internet in the previous 3 months in Q5 compared to 24% in Q1. Interestingly, monthly per capita expenditure exhibits an inverse pattern for outpatient demand compared to inpatient demand. Individuals in Q1 have a slightly higher average monthly expenditure of IDR 922,600, compared to IDR 885,300 in Q5.

These findings align with the heatmaps from the participation and consumption models (see C.4), although there are some differences in the most important effect modifiers. This suggests that the heterogeneity in the decision to access care and the quantity of care consumed is driven by distinct characteristics. Age is a potentially important effect modifier in the participation components of both care models, with a 19-year average age difference between Q5 and Q1 for inpatient participation (39.5 versus 20.5 years) and a 17-year difference for outpatient participation (41.2 versus 24.1 years). By contrast, socioeconomic factors emerge as stronger drivers of impact heterogeneity in the consumption components. For instance, in the inpatient consumption model, the disparity in household expenditure between Q5 and Q1 is more pronounced (IDR 1,154,600 versus IDR 778,000, a difference of IDR 376,600) compared to the participation model (IDR 895,000 versus IDR 778,000, a difference of IDR 117,000). Similarly, substantial variation exists in technology ownership and usage across quintiles, particularly for the consumption components. For outpatient consumption, internet usage shows a 16 percentage point difference between Q1 and Q5 (24% versus 8%), while for inpatient consumption, cellphone usage displays a 12% gap (57% versus 45%). These patterns highlight how different sociodemographic and economic factors influence the two distinct aspects of healthcare utilisation—the initial decision to seek care versus the intensity of services consumed once care is accessed.

#### 3.3.3 Drivers of heterogeneity: Univariable subgroup analysis.

We use the heatmaps from Fig 3 to identify additional data-driven effect modifiers not initially specified, focusing on covariates with large differences between Q1 and Q5 quintile means: marital status, employment status, household amenities (e.g. electricity), education, literacy, and technology (e.g. internet) usage. Table C.4 confirms these modifiers through variable importance outputs from the causal forest, with age and consumption expenditure consistently ranking as primary drivers of heterogeneity (importance values of 0.162-0.224 for age across models). However, we note that this analysis has limitations as forest-based algorithms may favour continuous variables due to their broader information range.

**Fig 4:**
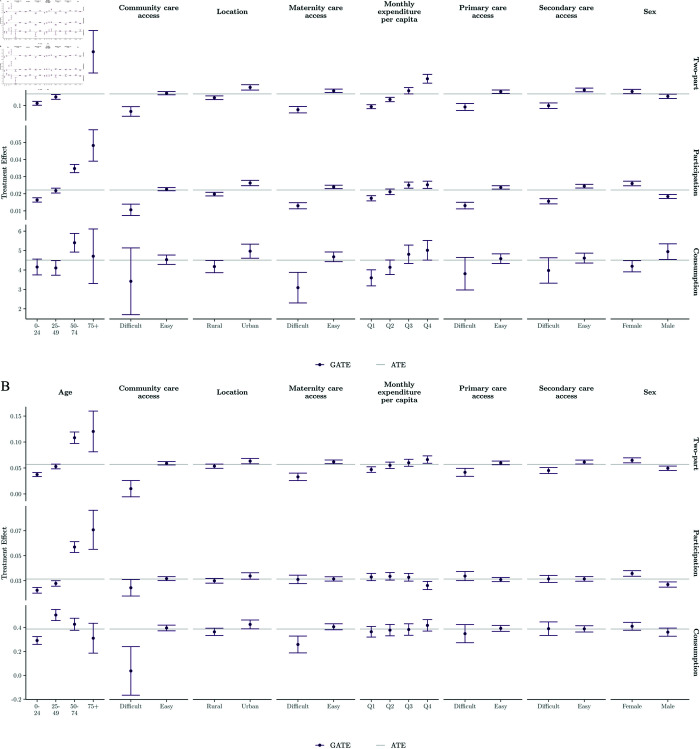
Estimated GATEs for theory-driven effect modifiers. (a) Inpatient demand. *Note:* Point estimates and 95% confidence intervals (error bars) are reported. ATE point estimates are also reported. (b) Outpatient demand. *Note:* Point estimates and 95% confidence intervals (error bars) are reported. ATE point estimates are also reported.

**Fig 5:**
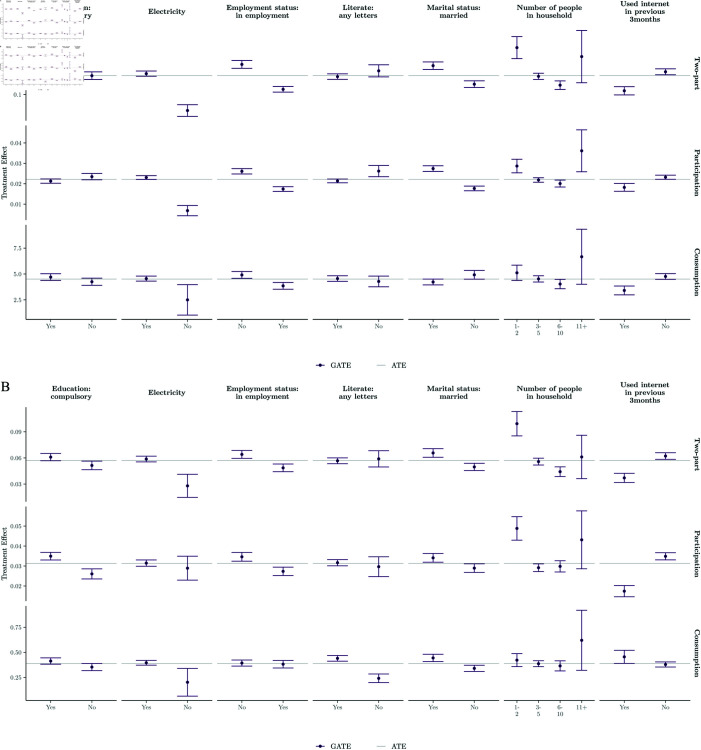
Estimated GATEs for data-driven effect modifiers. (a) Inpatient demand. *Note:* Point estimates and 95% confidence intervals (error bars) are reported. ATE point estimates are also reported. (b) Outpatient demand. *Note:* Point estimates and 95% confidence intervals (error bars) are reported. ATE point estimates are also reported.

Figs 4 and 5 present GATEs for theory-driven and data-driven modifiers, providing insights into heterogeneous insurance impacts. Notably, while older individuals generally exhibit increased demand for healthcare under insurance, this trend varies across different age groups, particularly in the context of consumption effects, where the greatest increase in outpatient visits occurs among individuals aged 25-49. Additionally, although treatment effects typically positively correlate with higher household wealth, the participation effect on outpatient demand is more pronounced among poorer households.

Furthermore, our findings suggest that policy effects are stronger for women across all models, except in the inpatient consumption model. Expectedly, regions with easy access to healthcare experience increased demand due to insurance coverage. However, disparities in treatment effects between areas with easy and difficult access underscore the need for aligning demand- and supply-side policies.

Moreover, our data-driven results reaffirm the positive association between socioeconomic status and treatment effects. Respondents with basic household amenities, such as electricity, tend to increase their consumption of healthcare in response to health insurance. Interestingly, technology usage emerges as a key effect modifier; however, individuals who use the internet are generally less likely to increase their healthcare demand compared to non-users, across all models except the participation component for outpatient demand.

Furthermore, married respondents and those out of work due to unemployment or retirement tend to increase their consumption of healthcare more as a result of being insured, a trend likely correlated with age. Additionally, being literate and educated is associated with a relative increase in inpatient care but a decrease in outpatient care compared to illiterate and uneducated individuals. Finally, very small (1-2 members) or very large (11+ members) households also exhibit larger treatment effects, with larger households showing a greater increase in the quantity of healthcare consumed.

#### 3.3.4 Drivers of heterogeneity: Multivariable BLP analysis.

**Fig 6:**
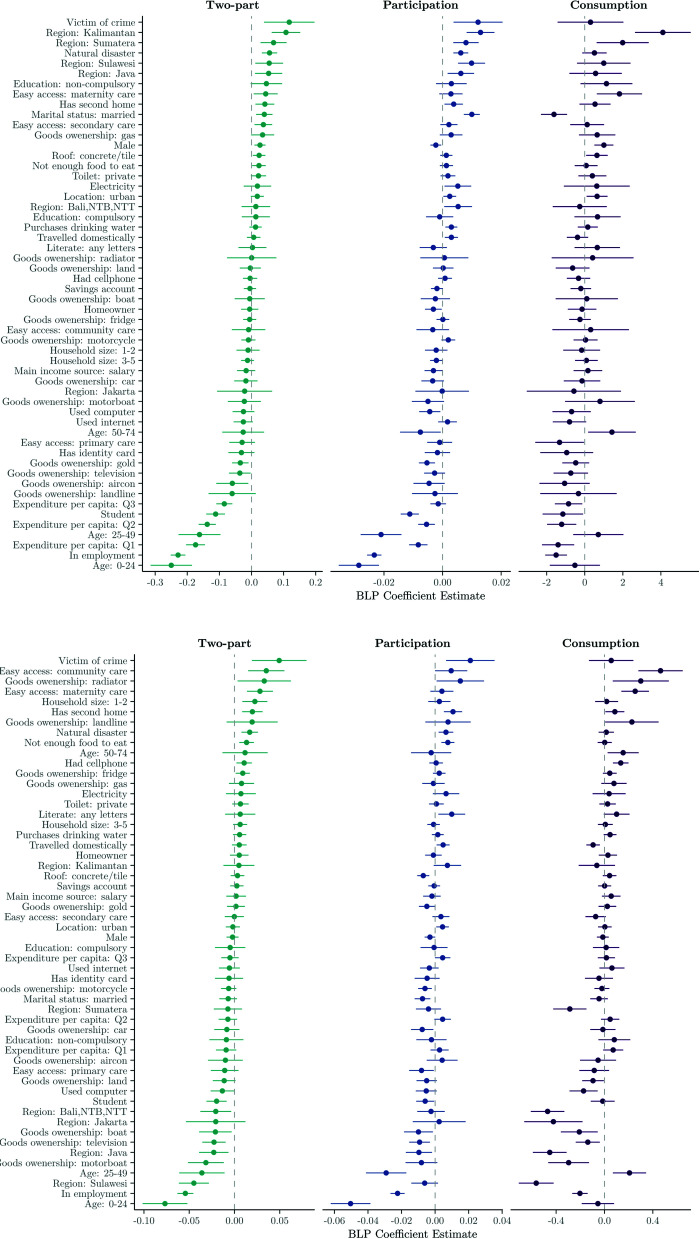
Estimated coefficients from the Best Linear Projection (BLP) of $\Gamma_{i}$ on $X_{i}$. (a) Inpatient demand. *Note:* BLP coefficients $\hat{\beta }$ from an OLS regression of ${\hat{\Gamma }}_{i}$ on *X*_*i*_ (${\hat{\Gamma }}_{i}={\hat{\beta }}_{0}\;+\;X\hat{\beta }$). Continuous variables have been converted to discrete variables. Reference categories include: Age 75+, Per capita expenditure: Q4, Region: Maluku-Papua and Household size: 11+. All other variables have a binary interpretation. (b) Outpatient demand. *Note:* BLP coefficients $\hat{\beta }$ from an OLS regression of ${\hat{\Gamma }}_{i}$ on *X*_*i*_ (${\hat{\Gamma }}_{i}={\hat{\beta }}_{0}\;+\;X\hat{\beta }$). Continuous variables have been converted to discrete variables. Reference categories include: Age 75+, Per capita expenditure: Q4, Region: Maluku-Papua and Household size: 11+. All other variables have a binary interpretation.

Figs 6 and C.5 illustrate the coefficients obtained from BLP analysis, derived from OLS regressions of doubly robust scores on both full and restricted covariate vectors. To recap, the restricted vector includes both theory-driven effect modifiers (i.e. age, sex, geographic location, monthly household expenditure per capita, and access to healthcare facilities) and data-driven effect modifiers (i.e. marital status, education, employment status, household size, electricity, and internet usage) identified through our heterogeneity analysis.

Upon analysis of the full covariate vector, our findings reinforce prior subgroup analyses, emphasising the significance of age, healthcare accessibility and socioeconomic factors in explaining treatment effect heterogeneity. In the inpatient model, being aged 65+ (compared to under 25) is associated with a 0.09-point increase in the estimated treatment effect. Living in Java increases expected effects by approximately 0.10, while having easy access to community care adds around 0.06. Urban residence and mid-range expenditure (Q3) also have positive coefficients, while being in the lowest expenditure quartile (Q1) decreases the expected treatment effect by roughly 0.07. In the outpatient participation model, easy access to primary care strongly predicts larger effects, with a coefficient near 0.09. Older age again plays a role (0.08 for 65+), as do regional and behavioural factors—e.g., internet usage (+0.03), and living in Sumatera (+0.04). Conversely, being male or in lower expenditure quartiles predicts smaller effects (–0.02 to –0.06).

When the covariate vector is restricted to theory- and data-driven effect modifiers, the direction of effects remains consistent with the full model, while some magnitudes become more pronounced. For inpatient care, individuals aged 50–74 show an increase of approximately 0.07 in expected treatment effect compared to those under 25, while easy access to community care is associated with an increase of around 0.06. In the outpatient model, the strongest predictor remains ease of access to primary care, with a coefficient close to 0.08. Being in the lowest expenditure quartile continues to predict lower treatment effects (–0.05), while urban residence and older age both contribute positively. These results reinforce the role of accessibility, age, and socioeconomic status in explaining heterogeneity in treatment effects, validating the variables selected for inclusion in the restricted model.

These findings warrant further investigation into how specific variables shape heterogeneity in treatment effects. Understanding these dynamics can help policymakers tailor interventions more effectively, ensuring that health insurance benefits are maximised across different segments of the population, thereby enhancing the overall equity and efficacy of the healthcare system.

## 4 Discussion

In this paper, we evaluated the average and heterogeneous impacts of Indonesia’s subsidised JKN programme on health care utilisation three years post-implementation. Our findings align with previous evaluations [27,60], confirming that subsidised insurance increases healthcare utilisation on average. Through separate evaluations on inpatient and outpatient demand, we found parallels with [27], noting a limited participation effect of subsidised health care. Unlike higher-income countries, where demand inducement typically leads to increased consumption, lower-income countries like Indonesia face additional challenges, such as time and transport costs or inadequate health facilities, which may diminish the incentive-based effects of insurance [37]. However, we did observe greater consumption effects of insurance, indicating an increase in the intensity of health care consumed among those who choose to access it.

Our analysis revealed substantial evidence of heterogeneity in treatment effects across population subgroups. Notable, 20% of the population, experienced negative participation effects for outpatient care, contradicting demand inducement theory. This finding contributes to limited existing evidence on minimal or adverse health effects of health insurance [16,25,28], particularly for utilisation outcomes [59]. By increasing the affordability of essential care, insurance may prompt a substitution effect between inpatient and outpatient demand, with insured individuals potentially bypassing outpatient services in favour of more comprehensive inpatient care when facing serious health concerns. This interpretation is supported by our observation of larger consumption effects on inpatient care and aligns with substitution patterns documented in other resource-constrained healthcare systems.

The two-part model used in our study offers important important methodological advantages by separating the decision to seek care (participation) from the intensity of services used (consumption). This distinction is particularly relevant in Indonesia’s context where different barriers affect each stage of care-seeking [50]. Our findings demonstrate that these components are influenced by different factors and respond differently to insurance coverage, suggesting that policy interventions may need to be tailored to address specific barriers at each stage of the care-seeking process. This aligns with the work by [58] and [24], who emphasise the importance of distinguishing between these decision processes when evaluating health policies.

Consistent with previous evaluations of JKN, we found stronger policy effects among respondents living in urban areas with better healthcare access [27]. However, supply-side constraints continue to limit the effectiveness of health financing policies, particularly in rural settings [1]. This urban-rural disparity reflects structural inequities in health system development that persist across many low- and middle-income countries [13,45]. The differential impact suggests that demand-side financing alone may be insufficient to overcome these structural inequities, highlighting the need for complementary supply-side interventions, particularly in rural areas where healthcare infrastructure remains limited.

Age emerged as a significant predictor of treatment effect heterogeneity in our analysis, with older individuals generally experiencing larger benefits from insurance coverage. This finding aligns with the health capital model proposed by [32], which suggests that health investment incentives increase with age as health capital depreciates. It also resonates with empirical work by [29] and [15], who found larger effects of insurance among older populations in the United States. In Indonesia’s context, the greater impact among older populations likely reflects both higher healthcare needs and greater financial constraints, making insurance particularly valuable for this demographic. This suggests potential value in targeted support mechanisms for older populations to maximise JKN’s impact.

Socioeconomic factors substantially influenced the magnitude of treatment effects, with stronger effects among individuals with higher socioeconomic status, as measured by household expenditure, education, and access to amenities. This pattern suggests that while JKN reduces financial barriers, other barriers persist for disadvantaged groups, including limited knowledge about entitlements, transportation challenges, opportunity costs of seeking care, and possibly discrimination within healthcare settings. Similar patterns observed elsewhere reflect the “inverse equity hypothesis" [33,65], raising important questions about universal coverage policies’ equity implications and highlighting the need for approaches specifically targeting vulnerable populations.

Our data-driven analysis identified additional important effect modifiers including marital status, employment status, and technology usage. Married individuals and those outside the workforce consumed more healthcare, likely reflecting age effects, time availability, and family support structures. The inverse relationship between technology usage and healthcare demand increase warrants further investigation – it may reflect better health literacy and preventive behaviour among technology users, or indicate technology access serves as a proxy for unmeasured factors influencing healthcare-seeking behaviour.

We demonstrated the potential of integrating machine learning into prediction and causal inference tasks, emphasising ensemble machine learning as a promising alternative to parametric regression for estimating nuisance parameters. While ensemble methods are sometimes considered “black-box" models due to their lack of interpretability, they are particularly well-suited for our objective of supporting the estimation of causal parameters. Ensemble methods effectively combine the strengths of diverse learners, minimising model misspecification and improving out-of-sample prediction performance. Their flexibility make them ideal for predicting nuisance parameters, especially when researchers have sufficient knowledge to make informed selections of the covariate base learner libraries and tuning parameters [53,55]. To enhance transparency, we report the weights assigned to each candidate learner in the superlearner ensemble, providing insight into the relative contribution of different algorithms to the final predictions. Moreover, their integration with causal inference techniques, such as causal forests, allows for accurate estimation of CATEs, even in settings with numerous covariates [61]. Importantly, while the internal mechanics of these algorithms may lack interpretability, the subsequent analyses—such as subgroup analysis, classification analysis, and best linear projection—offer valuable policy-relevant insights into treatment effect heterogeneity. This approach effectively bridges the gap between predictive accuracy and interpretability, enabling us to identify key drivers of heterogeneous impacts while maintaining robust causal estimates.

The study findings establish a foundation for examining complex policy-modifier interactions. While our analysis used binary covariates for clarity, real-world targeting requires considering multiple covariate combinations simultaneously. This has prompted research on “optimal policy rules" linking individual characteristics to treatment decisions [9,10,39–41,43,48,49,68]. For programmes like JKN, such rules could prioritise enrolment for those likely to benefit most while balancing equity considerations and budget constraints. This approach aligns with universal health coverage principles by promoting both efficient resource allocation and equitable implementation based on the heterogeneity patterns we’ve identified.

Our study has several limitations. First, we rely on a selection on observables framework, which assumes unconfoundedness. Despite including an extensive set of covariates, unobserved confounding may persist, particularly selection bias where individuals may self-select into insurance based on healthcare needs. This selection issue, well-documented in health insurance research [20,26], remains a challenge for observational studies. Future research could incorporate methods for CATE estimation that address unobserved confounding, such as instrumental variables or panel data approaches [8,69–71]. Second, while ensemble methods offer advantages for predicting nuisance parameters, our implementation prioritised simplicity over exhaustive optimisation. Though causal forests provide some robustness to misspecification through orthogonalisation, more refined model selection and validation could strengthen our analysis. Third, our cross-sectional data limits examination of temporal dynamics and longer-term effects of JKN. This prevents analysis of adaptation processes in healthcare-seeking behaviour over time. Additionally, our focus on utilisation excludes other important dimensions such as quality of care, efficiency, and patient satisfaction [22,45], pointing to important areas for future research. Finally, we focus exclusively on demand-side effects without modelling supply-side responses. Indonesia’s healthcare provision is characterised by significant supply constraints and quality variations [1,62], which may interact with insurance effects in complex ways. A more comprehensive evaluation would integrate both demand and supply considerations, examining how supply constraints moderate insurance effects across diverse geographic and socioeconomic contexts [30,47].

Despite these limitations, our study provides valuable insights for JKN refinement and a template for evaluating complex health system interventions.

## 5 Conclusion

This study evaluated Indonesia’s subsidised JKN health insurance program’s impact on healthcare utilisation, examining both average effects and heterogeneity across population subgroups. Our findings demonstrate that while JKN has successfully increased overall healthcare utilisation, the magnitude and nature of these effects vary substantially across different segments of the population.

The primary results of our analysis can be summarised as follows: Firstly, JKN implementation has led to increased utilisation of both outpatient and inpatient services on average, with more pronounced effects on the intensity of care consumed (consumption) than on the initial decision to seek care (participation). Secondly, substantial heterogeneity exists in the program’s impact, with more significant benefits observed among older individuals, those with superior access to healthcare facilities, and populations of higher socioeconomic status. Thirdly, approximately 20% of the population—predominantly those with limited healthcare access and lower socioeconomic status—experienced negligible or negative effects on outpatient participation, suggesting potential substitution between different modalities of care.

These findings have important policy implications for JKN and similar health insurance programs in developing countries. First, complementary supply-side interventions are needed to address persistent geographic inequities in healthcare access. In areas with limited healthcare infrastructure, expanding insurance coverage alone is insufficient to improve utilisation; investments in healthcare facilities, particularly in rural and underserved areas, would enhance JKN’s effectiveness by ensuring increased financial access translates into actual service utilisation. Second, targeted approaches should reach vulnerable populations who currently derive smaller benefits from JKN. This could include educational campaigns to improve health literacy, transportation subsidies to reduce geographic barriers, or outreach programs for underserved communities. Third, the stronger consumption effects compared to participation effects suggest JKN more effectively reduces financial burdens for those already accessing care than encourages initial care-seeking, pointing to the importance of addressing both financial and non-financial barriers including cultural factors, time constraints, and information asymmetries. Fourth, monitoring approaches should examine how benefits are distributed across the population beyond average effects to identify gaps in coverage and effectiveness.

Several significant challenges remain for future research and policy development. First, developing optimal policy rules represents a promising direction for future work. These rules could help target subsidies or complementary interventions to those most likely to benefit, enhancing the cost-effectiveness and equity of universal health coverage programs. By leveraging the heterogeneity patterns identified in our analysis, policymakers could design more efficient allocation mechanisms that prioritise resources toward population segments with the highest expected benefits while maintaining equity objectives. This would move beyond identifying heterogeneous effects to actively using this information for more dynamic and responsive policy frameworks. Second, addressing methodological challenges in causal inference for health policy evaluation remains important. While our causal machine learning approach offers advantages over traditional techniques, further methodological innovations are needed to address unobserved confounding and dynamic treatment effects. Third, expanding focus beyond utilisation to examine impacts on health outcomes, financial protection, and system efficiency represents an important frontier for JKN evaluation. Understanding whether increased utilisation translates into improved health and wellbeing is crucial for assessing universal health coverage initiatives’ ultimate success. Fourth, exploring interactions between demand-side financing mechanisms and supply-side factors such as provider incentives and healthcare quality would provide a more comprehensive understanding of health system dynamics. Finally, longitudinal studies tracking JKN’s long-term impacts will be essential as the program evolves, particularly as coverage expands and the healthcare system develops.

In conclusion, Indonesia’s JKN program has made significant strides toward increasing healthcare utilisation, but important disparities remain in who benefits from this expansion. By addressing the challenges identified in this study through targeted interventions, supply-side investments, and continued monitoring of heterogeneous effects, Indonesia can enhance the equity and effectiveness of its ambitious universal health coverage initiative, bringing quality healthcare within reach for all citizens regardless of their socioeconomic status or geographic location.

## Appendices

## A List of all covariates used for confounder adjustment

**Household member-level (binary)**

Male

Female

Marital status: single

Marital status: married

Marital status: divorced

Marital status: widow(er)

Has a national identity number

Literacy: Latin letters

Literacy: Arabic letters

Literacy: other letters

Education: compulsory

Education: non-compulsory

Travelled domestically for tourism in 2016

Victim of crime between March 2016-February 2017

Had a cellphone in previous 3 months

Used a computer in previous 3 months

Used internet in previous 3 months

Employment status: in employment

Employment status: student

Employment status: housekeeper

Employment status: out of employment

Employment status: performs other activities

**Household member-level (continuous)**

Age

**Household-level (binary)**

Location: urban

Not enough food to eat in previous year

Home occupancy status: owner

Home occupancy status: renter

Home occupancy status: rent-free

Home occupancy status: company-owned

Home occupancy status: other

Has a second home

Roof: concrete

Roof: tile

Roof: asbestos

Roof: zinc

Roof: bamboo

Roof: wood/shingle

Roof: straw/fiber/leaves/metroxylon sagu

Toilet: private

Toilet: shared

Toilet: none

Drinking water: bottled

Drinking water: tap

Drinking water: pump

Drinking water: protected well

Drinking water: unprotected well

Drinking water: protected spring

Drinking water: unprotected spring

Drinking water: river

Drinking water: rain

Drinking water: other

Purchases drinking water

Electricity

Natural disaster in previous year

Natural tourism in residential area

Savings account

Goods ownership: gas (over 5.5kg)

Goods ownership: fridge

Goods ownership: air conditioning

Goods ownership: radiator

Goods ownership: landline

Goods ownership: gold (over 10g)

Goods ownership: motorcycle

Goods ownership: boat

Goods ownership: motorboat

Goods ownership: car

Goods ownership: television

Goods ownership: land

Main income source: salary

Main income source: money transfer

Main income source: investments

Main income source: pension

**Household-level (continuous)**

Number of people in household

Number of children in household

Number of households in census building/house

Number of families in census building/house

Number of rooms in census building/house

Monthly consumption expenditure per capita (IDR 100,000)

**District-level (binary)**

Easy access: primary care

Easy access: community care

Easy access: maternal care

Easy access: secondary care

**Regional-level (binary)**

Region: Sumatera

Region: Jakarta

Region: Java

Region: Bali, NTB, NTT

Region: Kalimantan

Region: Sulawesi

Region: Maluka-Papua

## B Causal forests

One distinctive feature of causal forests is their use of an “honesty" concept. This process occurs at the individual “causal tree" level, where the dataset is divided into two equal parts: one for training the model and one for predicting treatment effects. Causal forests are comprised of many such causal trees, built using recursive partitioning, which systematically divides the data into smaller subsets to create a tree structure that captures the relationships between variables. The training subset initiates the splitting process by becoming the parent node.

Since optimising the loss function for all possible splits is computationally intensive, causal forests locally capture treatment effects using gradient-based optimisation. Pseudo-outcomes $\rho_{i}$ are computed for each observation in the parent node 𝒫, incorporating treatment, outcome, and propensity score estimates, as follows:


$$\rho_{i}=A_{𝒫}^{-1}(D_{i}-\hat{e}(X_{i})-{\bar{D}}_{𝒫})(Y_{i}-\hat{m}(X_{i})-{\bar{Y}}_{𝒫})-(D_{i}-\hat{e}(X_{i})-{\bar{D}}_{𝒫}){\hat{\beta }}_{𝒫}/{\text{Var}}_{𝒫}(D_{i}-\hat{e}(X_{i})),$$

$$A_{𝒫}=\frac{1}{I(i\in 𝒫)}\sum_{i\in 𝒫}(D-{\bar{D}}_{𝒫}{)}^{2},$$

where ${\bar{D}}_{P}$ and ${\bar{Y}}_{P}$ are the averages of *D*_*i*_ and *Y*_*i*_ over the observations in the parent node *P*, and ${\hat{\beta }}_{𝒫}$ is the solution of the least-squares regression of $Y_{i}\;-\;\hat{m}(X_{i})$ on $D_{i}\;-\;\hat{e}(X_{i})$ in 𝒫. These pseudo-outcomes guide the recursive partitioning process, aiming to maximise heterogeneity within each partition.

The recursive partitioning continues until terminal nodes contain a minimum number of treated and control observations, a threshold that can be optimized via cross-validation. Once the trees are fully grown, the prediction subset is used to assign weights to observations based on the proportion of times they fall into the same leaf as a given observation with the feature vector *X*_*i*_ = *x*. This process is repeated across all trees in the ensemble to obtain the final weighted estimates by averaging the weights assigned to each observation across all trees.

The grf package in R provides a variable_importance function, which calculates the importance of each covariate in *X*_*i*_. This measure is determined by considering the number of splits involving each covariate across all trees in the ensemble. Continuous variables tend to have higher importance compared to binary variables due to their potential for more splits.

Moreover, causal forests can incorporate sample weights into their model to ensure that treatment effect estimates represent the target population accurately. For Conditional Average Treatment Effects (CATEs), sample weights maintain the focus on the target population by minimising a weighted version of the loss function derived from the CATE estimator. However, for average treatment effects, sample weights alter the causal estimand, estimating the average effect over the population weighted by sample weights.

## C Additional figures/tables

**Table C.1:** Causal forest tuning parameters.

		Inpatient									Outpatient								
				Two-part			Participation			Consumption			Two-part			Participation			Consumption		
Tuning parameter	Description	$s_{1}$	$s_{2}$	$s_{3}$	$s_{1}$	$s_{2}$	$s_{3}$	$s_{1}$	$s_{2}$	$s_{3}$	$s_{1}$	$s_{2}$	$s_{3}$	$s_{1}$	$s_{2}$	$s_{3}$	$s_{1}$	$s_{2}$	$s_{3}$
sample.fraction	Fraction of the data used to build each tree	0.43	0.41	0.46	0.41	0.49	0.50	0.48	0.50	0.38	0.42	0.39	0.44	0.48	0.49	0.50	0.50	0.34	0.41
mtry	Number of variables tried for each split	22	29	27	23	25	26	27	28	17	24	24	28	23	25	26	30	25	22
min.node.size	Minimum number of observations in each tree leaf	4	2	2	1	1	1	1	1	1	1	1	2	1	1	1	5	2	1
honesty.fraction	Fraction of data used for determining splits	0.72	0.74	0.71	0.75	0.73	0.75	0.63	0.65	0.67	0.78	0.60	0.75	0.67	0.73	0.75	0.50	0.57	0.77
honesty.prune.leaves	Prunes estimation sample tree such that no leaves are empty	0	1	1	1	1	0	1	1	1	0	1	0	1	1	0	1	0	0
alpha	Maximum imbalance of a split	0.01	0.00	0.00	0.03	0.04	0.04	0.07	0.04	0.00	0.04	0.04	0.12	0.05	0.04	0.04	0.05	0.08	0.13
imbalance.penalty	Controls how harshly imbalanced splits are penalised	1.43	0.27	0.37	0.44	0.47	0.06	2.14	0.17	0.29	0.90	0.35	1.38	0.30	0.47	0.06	0.00	0.76	0.28

*Note:* List of all tuning parameters that are tuned by cross-validation in the training of the causal forests. Values are reported for causal forests trained on each subsample $\left\{s_{1},s_{2},s_{3}\right\}$.

**Table C.2:** Estimated cross-validated loss and standardised coefficients from fitted super learners.

	Propensity score		Outcome regression							
			Inpatient				Outpatient			
			Participation		Consumption		Participation		Consumption	
Algorithm	Loss	Coef	Loss	Coef	Loss	Coef	Loss	Coef	Loss	Coef
ensemble	0.550	-	0.142	-	2.545	-	0.342	-	0.376	-
gbm	0.552	1.246	0.140	0.237	2.588	0.004	0.339	0.447	0.376	0.110
glm-b	0.655	0.000	0.143	0.045	-	-	0.347	0.039	-	-
glm-g	-	-	-	-	2.552	0.092	-	-	0.381	0.000
glm-nb	-	-	-	-	3.243	0.000	-	-	1.070	0.000
glm-p	-	-	-	-	2.596	0.000	-	-	0.382	0.019
lasso-b	0.656	0.000	0.143	0.081	-	-	0.347	0.053	-	-
lasso-g	-	-	-	-	2.566	0.000	-	-	0.388	0.000
lasso-nb	-	-	-	-	3.243	0.000	-	-	1.070	0.000
lasso-p	-	-	-	-	2.576	0.003	-	-	0.382	0.000
nn	0.656	0.000	0.161	0.000	2.578	0.022	0.361	0.027	0.388	0.013
rf	0.673	0.000	0.145	0.167	2.549	0.090	0.355	0.068	0.382	0.009

*Note:* All estimates are averaged across the three subsamples $\left\{s_{1},s_{2},s_{3}\right\}$. Cross-validated loss estimates (“Loss") are reported for the base learners and the ensemble learner. Loss functions for the binary-response and count-response models are log loss and mean squared error respectively. Standardised regression coefficients (“Coef") are reported from the OLS regression of the observed outcomes on the cross-validated predicted outcomes. They can be interpreted as the “importance" of each base learner in making a prediction in the ensemble. Note that we use ordinary least squares (OLS) as the meta-learner, which allows for weights that do not sum to one. This arises from the inclusion of a constant term in the OLS model and the absence of non-negativity constraints on the weights.

**Table C.3:** Results from heterogeneity test using difference-in-means estimator.

	Inpatient				Outpatient			
	Est	Std.Err	Unadj *p*-val	Adj *p*-val	Est	Std.Err	Unadj *p*-val	Adj *p*-val
**Two-part**								
Q2-Q1	−0.002	0.013	0.900	0.899	0.042	0.005	0.000	0.000
Q3-Q1	0.029	0.013	0.027	0.046	0.044	0.005	0.000	0.000
Q4-Q1	0.075	0.013	0.000	0.000	0.062	0.005	0.000	0.000
Q5-Q1	0.308	0.013	0.000	0.000	0.135	0.005	0.000	0.000
**Participation**								
Q2-Q1	0.000	0.001	0.892	0.893	0.031	0.002	0.000	0.000
Q3-Q1	0.005	0.001	0.001	0.001	0.045	0.002	0.000	0.000
Q4-Q1	0.014	0.001	0.000	0.000	0.060	0.002	0.000	0.000
Q5-Q1	0.033	0.001	0.000	0.000	0.100	0.002	0.000	0.000
**Consumption**								
Q2-Q1	−0.054	0.377	0.885	0.886	0.125	0.038	0.001	0.001
Q3-Q1	0.193	0.377	0.608	0.820	0.124	0.038	0.001	0.001
Q4-Q1	0.339	0.377	0.368	0.695	0.138	0.038	0.000	0.001
Q5-Q1	1.549	0.377	0.000	0.000	0.134	0.038	0.000	0.001

*Note:* Table reports estimates and standard errors of the differences in sorted GATEs (for quintiles of predicted CATEs) between the lowest quintile (Q1) and higher quintiles (Q2-Q5). Unadj *p*-val does not correct for multiple hypothesis testing. Adj *p*-val uses the Romano-Wolf procedure to correct for multiple hypothesis testing.

**Fig C.4:**
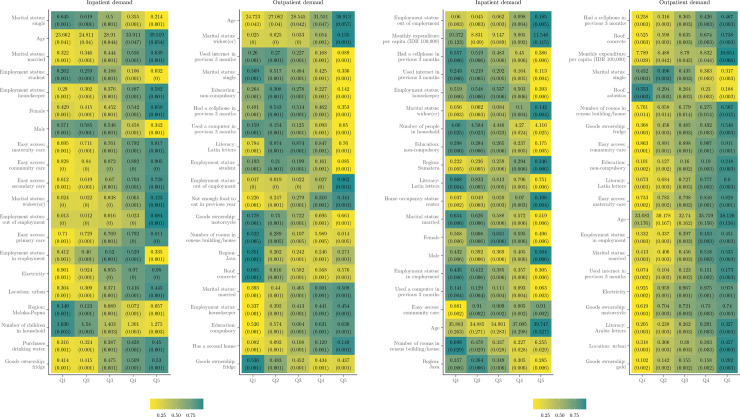
Heatmaps for classification analysis (participation and consumption models). (a) Participation model. *Note:* Heatmaps show the variation in the joint distribution of covariates for subgroups with different CATEs. The annotated text shows the average value and standard error (in parentheses) of each covariate within each quintile (of predicted CATEs). The colour is a normalised distance of each quintile-specific covariate mean from the overall covariate mean. The darker the colour, the greater the distance. Only the top 20 covariates in X are plotted, in descending order of variation between Q1 and Q5. (b) Consumption model. *Note:* Heatmaps show the variation in the joint distribution of covariates for subgroups with different CATEs. The annotated text shows the average value and standard error (in parentheses) of each covariate within each quintile (of predicted CATEs). The colour is a normalised distance of each quintile-specific covariate mean from the overall covariate mean. The darker the colour, the greater the distance. Only the top 20 covariates in X are plotted, in descending order of variation between Q1 and Q5.

**Table C.4:** Variable importance results from estimated causal forests.

**(a) Inpatient demand.**						
**Ranking**	**s1**		**s2**		**s3**	
	**Effect modifier**	**Importance**	**Effect modifier**	**Importance**	**Effect modifier**	**Importance**
1	Employment status: out of employment	0.182	Employment status: out of employment	0.226	Age	0.188
2	Age	0.174	Age	0.162	Employment status: out of employment	0.168
3	Monthly expenditure per capita (IDR 100,000)	0.091	Monthly expenditure per capita (IDR 100,000)	0.119	Monthly expenditure per capita (IDR 100,000)	0.104
4	Marital status: widow(er)	0.078	Number of rooms in census building/house	0.079	Number of people in household	0.080
5	Employment status: in employment	0.073	Marital status: single	0.041	Home occupancy status: company-owned	0.057
**Participation**						
1	Age	0.199	Marital status: single	0.160	Marital status: single	0.187
2	Employment status: in employment	0.092	Age	0.139	Age	0.171
3	Marital status: widow(er)	0.057	Marital status: married	0.075	Marital status: married	0.123
4	Marital status: single	0.054	Female	0.072	Employment status: student	0.061
5	Easy access: maternity care	0.052	Male	0.061	Easy access: secondary care	0.042
**Consumption**						
1	Easy access: secondary care	0.135	Monthly expenditure per capita (IDR 100,000)	0.153	Monthly expenditure per capita (IDR 100,000)	0.117
2	Monthly expenditure per capita (IDR 100,000)	0.112	Employment status: out of employment	0.077	Number of people in household	0.068
3	Easy access: primary care	0.088	Age	0.055	Age	0.066
4	Age	0.074	Home occupancy status: renter	0.054	Easy access: community care	0.061
5	Easy access: maternity care	0.053	Easy access: secondary care	0.053	Easy access: secondary care	0.058
*Note:* Top 5 important effect modifiers are reported based on the variable importance ranking from the trained causal forests fitted on each subsample $\left\{s_{1},s_{2},s_{3}\right\}$. Importance is measured as the weighted sum of the frequency with which the variable was used to split on at each depth in the forest.						
**(b) Outpatient demand.**						
**Ranking**	**s1**		**s2**		**s3**	
	**Effect modifier**	**Importance**	**Effect modifier**	**Importance**	**Effect modifier**	**Importance**
1	Age	0.162	Age	0.224	Age	0.217
2	Easy access: primary care	0.083	Marital status: widow(er)	0.090	Easy access: secondary care	0.087
3	Easy access: secondary care	0.079	Easy access: secondary care	0.072	Monthly expenditure per capita (IDR 100,000)	0.068
4	Marital status: widow(er)	0.073	Monthly expenditure per capita (IDR 100,000)	0.061	Marital status: single	0.059
5	Number of households in census building/house	0.073	Easy access: maternity care	0.059	Easy access: maternity care	0.054
**Participation**						
1	Age	0.178	Age	0.212	Age	0.230
2	Easy access: secondary care	0.131	Marital status: widow(er)	0.110	Marital status: single	0.068
3	Easy access: primary care	0.066	Monthly expenditure per capita (IDR 100,000)	0.078	Marital status: widow(er)	0.065
4	Easy access: maternity care	0.060	Easy access: secondary care	0.063	Easy access: secondary care	0.051
5	Monthly expenditure per capita (IDR 100,000)	0.059	Used internet in previous 3 months	0.059	Easy access: maternity care	0.041
**Consumption**						
1	Monthly expenditure per capita (IDR 100,000)	0.084	Monthly expenditure per capita (IDR 100,000)	0.130	Monthly expenditure per capita (IDR 100,000)	0.107
2	Easy access: community care	0.074	Easy access: maternity care	0.095	Easy access: secondary care	0.107
3	Age	0.071	Age	0.088	Easy access: primary care	0.096
4	Easy access: primary care	0.068	Easy access: secondary care	0.067	Easy access: maternity care	0.084
5	Easy access: maternity care	0.066	Easy access: primary care	0.059	Age	0.070
*Note:* Top 5 important effect modifiers are reported based on the variable importance ranking from the trained causal forests fitted on each subsample $\left\{s_{1},s_{2},s_{3}\right\}$. Importance is measured as the weighted sum of the frequency with which the variable was used to split on at each depth in the forest.						

**Fig C.5:**
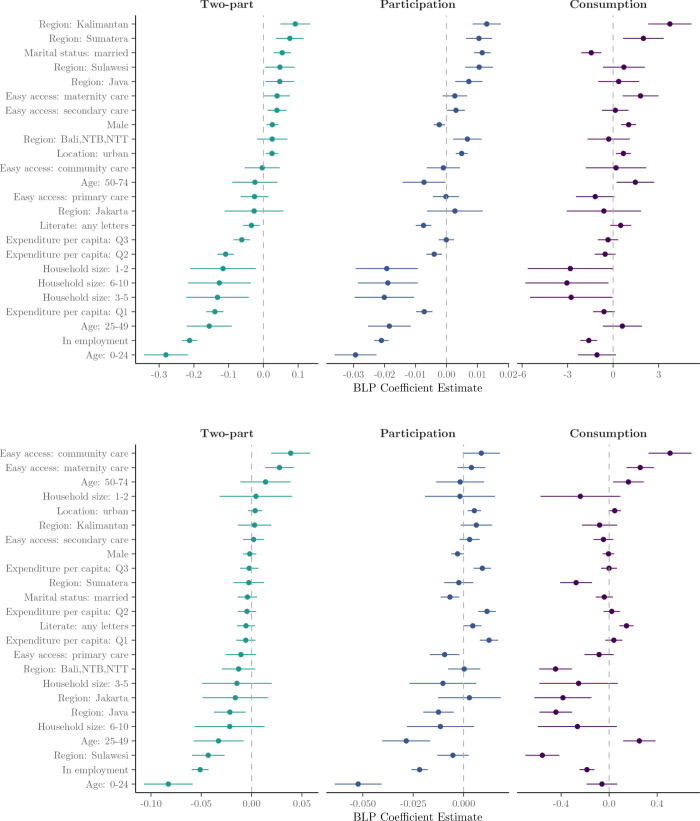
Estimated coefficients from the Best Linear Projection (BLP) of $\Gamma_{i}$ on $A_{i}\in X_{i}$. (a) Inpatient demand. *Note:* BLP coefficients $\hat{\beta }$ from an OLS regression of ${\hat{\Gamma }}_{i}$ on $A_{i}\in X_{i}$ (${\hat{\Gamma }}_{i}={\hat{\beta }}_{0}\;+\;A\hat{\beta }$). Continuous variables have been converted to discrete variables. Reference categories include: Age 75+, Per capita expenditure: Q4, Region: Maluku-Papua and Household size: 11+. All other variables have a binary interpretation. (b) Outpatient demand Note: BLP coefficients $\hat{\beta }$ from an OLS regression of ${\hat{\Gamma }}_{i}$ on $A_{i}\in X_{i}$ (${\hat{\Gamma }}_{i}={\hat{\beta }}_{0}\;+\;A\hat{\beta }$). Continuous variables have been converted to discrete variables. Reference categories include: Age 75+, Per capita expenditure: Q4, Region: Maluku-Papua and Household size: 11+. All other variables have a binary interpretation.
